# The Circadian Clock in Lepidoptera

**DOI:** 10.3389/fphys.2021.776826

**Published:** 2021-11-17

**Authors:** Daniel Brady, Alessio Saviane, Silvia Cappellozza, Federica Sandrelli

**Affiliations:** ^1^Department of Biology, Università di Padova, Padova, Italy; ^2^Council for Agricultural Research and Economics, Research Centre for Agriculture and Environment (CREA-AA), Padova, Italy

**Keywords:** Lepidoptera, circadian clock, circadian behaviors, larval circadian clock, pest insects, beneficial insects

## Abstract

With approximately 160,000 identified species of butterflies and moths, Lepidoptera are among the most species-rich and diverse insect orders. Lepidopteran insects have fundamental ecosystem functions as pollinators and valuable food sources for countless animals. Furthermore, Lepidoptera have a significant impact on the economy and global food security because many species in their larval stage are harmful pests of staple food crops. Moreover, domesticated species such as the silkworm *Bombyx mori* produce silk and silk byproducts that are utilized by the luxury textile, biomedical, and cosmetics sectors. Several Lepidoptera have been fundamental as model organisms for basic biological research, from formal genetics to evolutionary studies. Regarding chronobiology, in the 1970s, Truman’s seminal transplantation experiments on different lepidopteran species were the first to show that the circadian clock resides in the brain. With the implementation of molecular genetics, subsequent studies identified key differences in core components of the molecular circadian clock of Lepidoptera compared to the dipteran *Drosophila melanogaster*, the dominant insect species in chronobiological research. More recently, studies on the butterfly *Danaus plexippus* have been fundamental in characterizing the interplay between the circadian clock and navigation during the seasonal migration of this species. Moreover, the advent of Next Generation Omic technologies has resulted in the production of many publicly available datasets regarding circadian clocks in pest and beneficial Lepidoptera. This review presents an updated overview of the molecular and anatomical organization of the circadian clock in Lepidoptera. We report different behavioral circadian rhythms currently identified, focusing on the importance of the circadian clock in controlling developmental, mating and migration phenotypes. We then describe the ecological importance of circadian clocks detailing the complex interplay between the feeding behavior of these organisms and plants. Finally, we discuss how the characterization of these features could be useful in both pest control, and in optimizing rearing of beneficial Lepidoptera.

## Introduction

The daily rotation of the Earth causes predictable 24-h environmental cycles that have resulted in the evolution of circadian clocks, endogenously maintained timing mechanisms in almost all organisms, including bacteria, fungi, plants, and metazoan (Paranjpe and Sharma, 2005; Rosbash, 2009; Eelderink-Chen et al., 2021). These internal clocks are synchronized (entrained) by environmental stimuli, such as daylight or temperature, enabling organisms to adapt to environmental changes in phase with the 24-h day. Moreover, they regulate molecular, cellular, physiological, and behavioral rhythms, which show a periodicity of ~ 24h in the absence of any external cue (free-running condition). In insects, circadian clocks control the rhythmicity of several behaviors and physiological features, including egg-hatching, pupation, adult eclosion, locomotion, mating, feeding, and metabolism (Saunders, 2002; Tomioka and Matsumoto, 2010; Franco et al., 2018). The first circadian clock gene (*period*) was identified in *Drosophila melanogaster* (Konopka and Benzer, 1971); since then, the species has been the dominant model to elucidate the molecular genetic mechanisms underlying circadian rhythms in insects. However, studies have also been conducted in other insect species, many including Lepidoptera.

**Figure 1:**
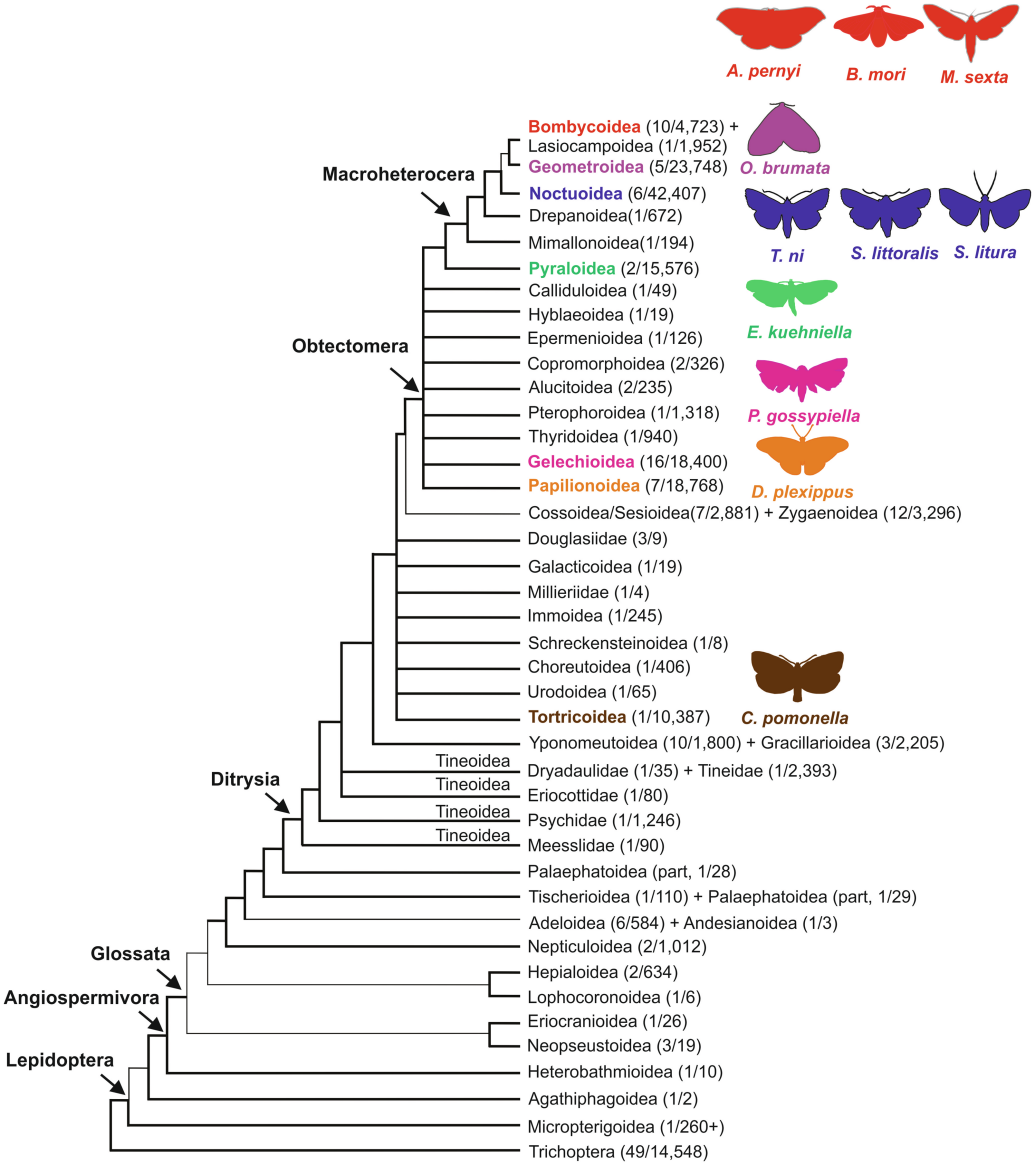
Phylogeny of Lepidoptera superfamilies, including Trichoptera, sister group to Lepidoptera (Mitter et al., 2017). Superfamilies, including species with at least one circadian phenotype described in this review, are reported with different colors: brown: Tortricoidea; orange: Papilionoidea; magenta: Gelechioidea; green: Pyraloidea; blue: Noctuoidea; purple: Geometroidea; and red: Bombycoidea. Representative species of each superfamily are shown using the same color code. As in Mitter et al. (2017), thicker lines indicate more strongly recognized groupings; numbers within parenthesis indicate the number of families/species. On the left, the following higher-level classification groups are shown: Angiospermivora, angiosperm feeding; Glossata, acquisition of the proboscis in adults; Ditrysia, a clade containing 98% of lepidopteran species; Obtectomera, a clade comprising butterflies and macro-moths; Macroheterocera, a clade of moths including superfamilies previously placed in the Macrolepidoptera group, which is currently not considered a monophyletic group (Mimallonoidea, Drepanoidea, Noctuoidea, Geometroidea, Lasiocampoidea, and Bombycoidea; Mitter et al., 2017). Modified from Mitter et al. (2017).

The order of Lepidoptera comprises butterflies and moths and is among the most species-rich and diverse insect groups. It includes approximately 160,000 identified species, subdivided into ~ 43–45 superfamilies (Mitter et al., 2017; Kawahara et al., 2019; Figure 1). Lepidoptera are holometabolous insects, showing juvenile stages (larvae or caterpillars) morphologically distinct from the mature adult form (moths and butterflies). The majority of Lepidoptera are nocturnal (about 75–85% of the identified species), while the remaining 15–25% of diurnal species also includes butterflies (Papilionoidea, ~19,000 described species; Kawahara et al., 2018). The overwhelming majority of Lepidoptera (>99%) are herbivores with over 90% having a host plant range of three or fewer species (Pierce, 1995); they are found in almost all terrestrial ecosystems and have fundamental ecosystem roles as pollinators and prey. They also have a significant impact on the human economy, as many species represent serious food crop pests, while others such as the domesticated silkworm *Bombyx mori* (Goldsmith et al., 2005) and *Antheraea pernyi* (Li et al., 2017) produce silk that is utilized by the textile industry. Moreover, silk is employed in innovative applications of both the biomedical and cosmetics sectors.

A recent phylogenomic analysis dated the most recent common ancestor of Lepidoptera in the late Carboniferous, about 300million years ago. Evolutionary studies support the hypothesis that Lepidoptera developed initially as internal feeders of non-vascular plants, then differentiated and coevolved in parallel with angiosperms (Figure 1). In this process, the larval stages became external plant feeders, while adults evolved a tube-like proboscis, used for the efficient acquisition of nutritious nectar from flowering plants (Mitter et al., 2017; Kawahara et al., 2019; Figure 1). These adaptations along with an increased ability to fly and colonize new plants are believed to be associated with the large body size observed in many moths and butterflies (Powell et al., 1998; Kawahara et al., 2019).

Several of the larger lepidopteran species have been model organisms in the study of different biological phenomena, including development, physiology, ecology, and evolution (Roe et al., 2009). Pioneering Lepidoptera-based investigations have been fundamental in the study of circadian biology, showing for the first time that the brain is the site of a light-entrainable circadian clock controlling behavioral rhythms (Williams, 1963; Truman and Riddiford, 1970).

This review presents an updated overview of the circadian clock in Lepidoptera. After a general description of the different behavioral circadian rhythms currently identified, we report the molecular and anatomical organization of circadian clocks in the adult stage and during development. We then describe the ecological importance of circadian clocks detailing the complex interplay between lepidopteran feeding behavior and plants. Finally, we discuss how the characterization of these features could be useful in both pest control and rearing improvements of beneficial Lepidoptera.

## Circadian Behavioral Rhythms From Embryo to Adulthood in Lepidoptera

From embryo to adulthood, both nocturnal and diurnal Lepidoptera exhibit multiple daily behavioral rhythms restricted to species-specific daytimes. Furthermore, for some species, these rhythmic activities have been demonstrated to be maintained under free-running conditions, indicating they are controlled by an endogenous circadian clock (Merlin and Reppert, 2009; Groot, 2014).

One of the first described circadian events occurring during lepidopteran development is egg-hatching, i.e., the emergence of the larva from the protective envelopes of the egg. In several moth species, egg-hatching occurs in the first hours of the day with a peak anticipating morning light-on, when maintained in light/dark (LD) entraining conditions (Minis and Pittendrigh, 1968; Sakamoto and Shimizu, 1994; Sauman and Reppert, 1998). This egg-hatching rhythm was demonstrated to be truly circadian as it was entrainable by environmental stimuli (such as light and/or temperature) from mid embryogenesis and was maintained in constant darkness (DD) regimes in the pink bollworm *Pectinophora gossypiella* (superfamily Gelechioidea; Minis and Pittendrigh, 1968) and the Chinese oak silk moth *Antheraea pernyi* (superfamily Bombycoidea; Sauman et al., 1996; Sauman and Reppert, 1998). Circadian egg-hatching rhythms have been described in other lepidopteran species, including the domesticated silkworm *B. mori* (superfamily Bombycoidea; Sakamoto and Shimizu, 1994; Table 1).

**Table 1:** Circadian behavioral rhythms in Lepidoptera^*^.

Circadian behavior	Superfamily	Family	Species	References
Egg-hatching	Gelechioidea	Gelechiidae	*Pectinophora gossypiella*	Minis and Pittendrigh, 1968
	Pyraloidea	Crambidae	*Diatraea grandiosella*	Takeda, 1983
	Bombycoidea	Saturniidae	*Antheraea pernyi*	Sauman et al., 1996; Sauman and Reppert, 1998
		Bombycidae	*Bombyx mori*	Sakamoto and Shimizu, 1994
Larval feeding	Noctuoidea	Noctuidae	*Trichoplusia ni*	Goodspeed et al., 2012
			*Spodoptera littoralis*	Suszczynska et al., 2017
			*Spodoptera litura*	Zhang et al., 2021
Larval locomotor activity	Noctuoidea	Noctuidae	*Spodoptera litura*	Zhang et al., 2021
Larval defecation	Noctuoidea	Noctuidae	*Spodoptera litura*	Zhang et al., 2021
Larval gut purging	Bombycoidea	Saturniidae	*Samia cynthia ricini*	Mizoguchi and Ishizaki, 1982
Adult eclosion	Pyraloidea	Crambidae	*Diatraea grandiosella*	Takeda, 1983
	Bombycoidea	Saturniidae	*Antheraea pernyi*	Truman and Riddiford, 1970
			*Hyalophora cecropia*	
		Bombycidae	*Bombyx mori*	Truman, 1972; Shimizu and Matsui, 1983
	Noctuoidea	Erebidae	*Hyphantria cunea*	Morris and Takeda, 1994
	Papillionoidea	Nymphalidae	*Danaus plexippus*	Froy et al., 2003
Adult locomotor activity or flight	Pyraloidea	Pyralidae	*Ephestia kuehniella*	Kobelková et al., 2015
	Bombycoidea	Saturniidae	*Antheraea pernyi*	Truman, 1974
			*Hyalophora cecropia*	
			*Samia cynthia ricini*	
		Sphingidae	*Manduca sexta*	Broadhead et al., 2017
			*Hyles lineata*	
Female calling behavior	Pyraloidea	Pyralidae	*Ephestia kuehniella*	Závodská et al., 2012
	Tortricoidea	Tortricidae	*Grapholita molesta*	Baker and Cardé, 1979
			*Cydia pomonella*	Castrovillo and Cardé, 1979
	Noctuoidea	Noctuidae	*Pseudaletia unipuncta*	Turgeon and McNeil, 1982; Delisle and McNeil, 1987
			*Helicoverpa assulta*	Kamimura and Tatsuki, 1993
			*Spodoptera littoralis*	Silvegren et al., 2005; Sadek et al., 2012
			*Agrotis segetum*	Rosén et al., 2003
Male attraction behavior	Tortricoidea	Tortricidae	*Cydia pomonella*	Castrovillo and Cardé, 1979
	Noctuoidea	Noctuidae	*Agrotis segetum*	Rosén et al., 2003
			*Trichoplusia ni*	Linn et al., 1996
			*Autographa californica* (weak?)	Shorey and Gaston, 1965
			*Heliothis virescens*	
			*Spodoptera exigua*	
			*Spodoptera littoralis*	Silvegren et al., 2005
Mating	Noctuoidea	Noctuidae	*Spodoptera littoralis*	Silvegren et al., 2005
Sun-compass orientation during migration	Papillionoidea	Nymphalidae	*Danaus plexippus*	Perez et al., 1997; Mouritsen and Frost, 2002; Froy et al., 2003; Merlin et al., 2009
		Pieridae	*Aphrissa statira*	Oliveira et al., 1998
			*Phoebis argante*	

* Data referring to behavioral rhythms evaluated in constant conditions.

During post-embryonic development, lepidopteran larvae are characterized by intense feeding and recent studies demonstrated that for some Lepidoptera, feeding behavior in the last larval instar occurs with a daily rhythm in entraining conditions (Goodspeed et al., 2012; Suszczynska et al., 2017; Niepoth et al., 2018; Zhang et al., 2021). Studies on different pest species belonging to the Noctuoidea superfamily, including the cabbage looper *Trichoplusia ni*, as well as the cotton leafworms *Spodoptera littoralis* and *Spodoptera litura*, analyzed feeding behavior in both LD and DD conditions. They indicated that in these species larval feeding is under circadian control (Goodspeed et al., 2012; Suszczynska et al., 2017; Zhang et al., 2021). Moreover, *S. litura* larvae showed rhythmic circadian locomotor and defecation activities, respectively peaking during the night and the day, in both LD and DD conditions (Zhang et al., 2021; Table 1). Additionally, gut purging, which occurs at the end of larval development to empty the gut before entering the pupal stage, was shown to be under circadian control in the silkmoth *Samia cynthia* var. *ricini* (superfamily Bombycoidea; Mizoguchi and Ishizaki, 1982; Table 1).

Adult eclosion, i.e., adult emergence from the pupal exuvia, is one of the most vulnerable events during an insect’s life cycle, and in many species is controlled by the circadian clock. Eclosion occurs at species-specific times of the day in different lepidopteran species when maintained in LD conditions and likely restricts the timing of this behavior to the most advantageous moments of the day (Merlin and Reppert, 2009). In constant conditions, adult eclosion maintains a 24-h rhythmic profile, as demonstrated for species belonging to the superfamilies of Bombycoidea (the giant silkmoth *Hyalophora cecropia*; Truman and Riddiford, 1970; *B. mori*; Truman, 1972; Shimizu and Matsui, 1983; *A. pernyi*; Truman and Riddiford, 1970), Noctuoidea (the fall webworm *Hyphantria cunea*; Morris and Takeda, 1994), Pyraloidea (the southwestern corn borer *Diatraea grandiosella*; Takeda, 1983), and Papilionoidea (the monarch butterfly *Danaus plexippus*; Froy et al., 2003; Table 1).

Adult flight is related to vital activities for both moths and butterflies, including nectar feeding and mating. To date, the circadian control of flight behavior has been evaluated in relatively few Lepidoptera. Nevertheless, a flight circadian rhythmicity has been demonstrated in species belonging to the superfamily Bombycoidea, initially in *Hyalophora cecropia*, *S. cynthia* var. *ricini*, *A. pernyi* (Truman, 1974), and more recently in the hawkmoths *Manduca sexta* and *Hyles lineata* (Broadhead et al., 2017). In LD conditions, the nocturnal moths *H. cecropia*, *S. cynthia* var. *ricini, A. pernyi*, and *M. sexta* display rhythmic flight restricted to the dark period of the day, while the crepuscular *H. lineata* shows a bimodal activity, with a first peak after light-on and a second after light-off. In DD, all these species maintain 24h rhythmic flight, although with some modifications in their profiles (Truman, 1974; Broadhead et al., 2017; Table 1).

In moths, several behavioral activities related to reproduction have been shown to have a rhythmic daily species-specific pattern (reviewed in Groot, 2014). In these species, reproduction is characterized by stereotypical behaviors anticipating actual mating, associated with the production and/or release of sex pheromones by females (reviewed in Groot, 2014; Stepien et al., 2020). During sex pheromone emission, females adopt typical postures (named calling), characterized by a modification of the abdomen position and the extrusion of the pheromone gland to better disperse sex pheromones in the environment (Stepien et al., 2020). Males perceive sex pheromones through the antennae and fly toward the female for mating (Stepien et al., 2020). In several moths, female calling, male response to female sex pheromones, and mating have been shown to have a rhythmic daily species-specific pattern in LD conditions (Groot, 2014). Some studies examined these behaviors under constant conditions, demonstrating a circadian control of these daily activities. For example, female calling behavior was shown to maintain its rhythmicity in DD in species of the superfamily Noctuoidea (the true armyworm moth *Pseudaletia unipuncta*, the oriental tobacco budworm moth *Helicoverpa assulta*, and *S. littoralis*; Turgeon and McNeil, 1982; Delisle and McNeil, 1987; Kamimura and Tatsuki, 1993; Silvegren et al., 2005; Sadek et al., 2012), in some Tortricoidea (the oriental fruit moth *Grapholita molesta* and the codling moth *Cydia pomonella*; Baker and Cardé, 1979; Castrovillo and Cardé, 1979), and in the Mediterranean flour moth *Ephestia kuehniella* (superfamily Pyraloidea; Závodská et al., 2012; Table 1). A 24h rhythmic male response to female sex pheromones was demonstrated in constant conditions in some Noctuoidea moths (the turnip moth *Agrotis segetum*, *T. ni*, *Heliothis virescens*, *Spodoptera exigua*, and *S. littoralis*; Shorey and Gaston, 1965; Castrovillo and Cardé, 1979; Linn et al., 1996; Rosén et al., 2003; Silvegren et al., 2005) and in the Tortricoidea *C. pomonella* (Castrovillo and Cardé, 1979; Table 1). A 24h rhythm was detected in mating frequency of *S. littoralis* in both 12:12 LD and DD conditions (Silvegren et al., 2005). Additionally, in *S. littoralis*, optimized mating efficiency requires synchronized circadian clocks in phase between the two sexes, as a decreased frequency in mating was detected when males and females possessed out of phase circadian clocks (Silvegren et al., 2005).

Various lepidopteran species show a migratory behavior due to seasonally changing environments (Williams, 1957; Walker, 1980). Migration has been extensively studied in the eastern North American populations of the monarch butterfly *D. plexippus*, which are characterized by oriented flight and each Autumn can travel up to 4,500km to reach wintering sites in central Mexico. Migrants traveling southward are characterized by a reproductive diapause and enhanced longevity. In Spring, adults exit from reproductive diapause, mate, and remigrate to the north, with females laying fertilized eggs in the southern United States. The progeny of these butterflies continues migrating North, reaching the original sites in North America, in a mostly multigenerational migratory phenomenon (Perez et al., 1997; recently reviewed in Reppert et al., 2016; Reppert and de Roode, 2018; Merlin et al., 2020). To generate accurate orientation during migration, monarchs possess well-characterized sun and magnetic compasses, which incorporate daylight cues and the Earth’s magnetic field information, respectively (Mouritsen and Frost, 2002; Froy et al., 2003; Guerra et al., 2014). The sun compass requires the circadian clock to compensate for the apparent movement of the sun in the sky during the day (Mouritsen and Frost, 2002; Froy et al., 2003; Merlin et al., 2009). Additionally, a specific molecular clock element with a clock-independent role (DpCRYPTOCHROME1, DpCRY1) is involved in the proper functioning of the magnetic compass (Gegear et al., 2008, 2010; Wan et al., 2021). As the role of DpCRY1 in *D. plexippus* magnetoreception is not related to its function in the circadian clock, this aspect is not further discussed in this review (for interested readers: Reppert et al., 2016; Reppert and de Roode, 2018; Merlin et al., 2020; Wan et al., 2021).

The involvement of the circadian clock for time-compensated navigation during migration has been hypothesized in other butterflies such as *Aphrissa statira* and *Phoebis argante* (Oliveira et al., 1998; Table 1). Migration has great relevance for the functionality of an ecosystem since newly arrived migrants can have a role as pollinators, representing a food source for insectivores and in turn for the other components of a trophic system. As migration is typical of several butterflies and moths (Williams, 1957), it would be interesting to understand whether the circadian clock is involved in orientation during navigation of other lepidopteran species.

## The Circadian System in Lepidoptera

### The Molecular Circadian Clock

**Figure 2:**
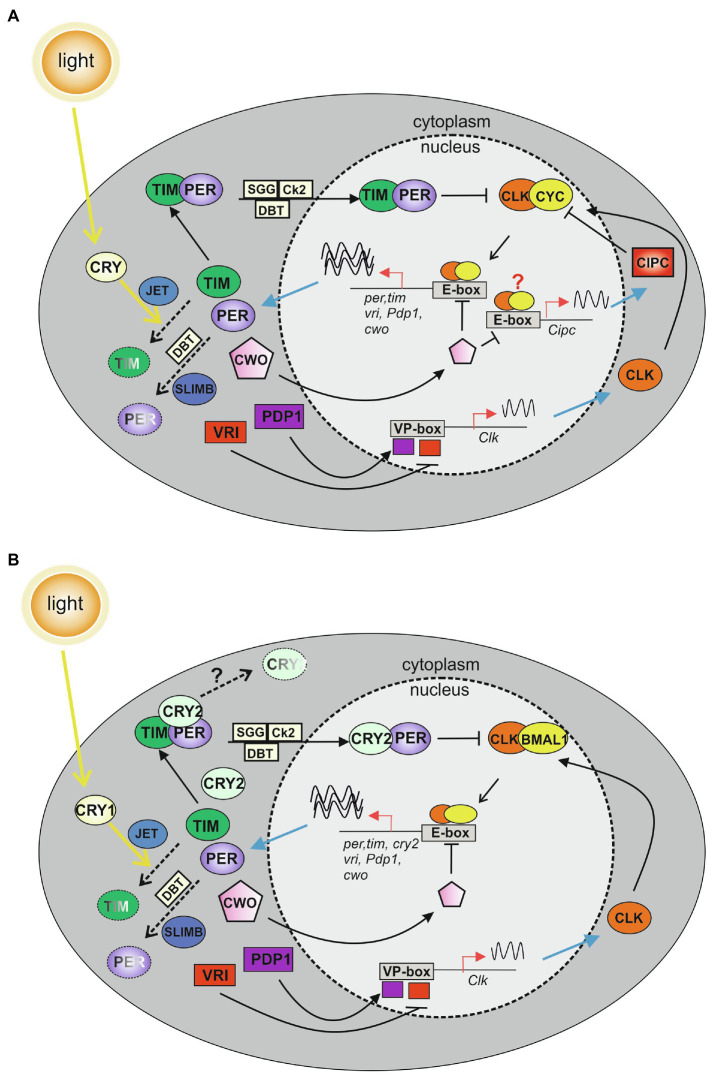
Model of the major transcription–translation feedback loops (TTLs) in the circadian molecular clock of *Drosophila melanogaster* **(A)** and Lepidoptera **(B)**. **(A)** In the first TTL of *D. melanogaster*, CLOCK (CLK) and CYCLE (CYC) dimerize and bind to E-boxes in the promoters of *per* and *tim* clock genes. PERIOD (PER) and TIMELESS (TIM) enter the nucleus as a complex, and inhibit CLK-CYC activity (Allada et al., 1998; Bae et al., 1998; Darlington et al., 1998; Rutila et al., 1998; Glossop et al., 1999; Lee et al., 1999). A second TTL modulates *Clk* expression: CLK:CYC dimers induce the transcription of *vrille* (*vri*) and *par domain protein 1* (*Pdp1*) genes. VRI and PDP1 compete for the same element (VP-box) in the *Clk* promoter, controlling *Clk* transcription (Cyran et al., 2003). In the third TTL, CLK:CYC dimers activate the transcription of *clockwork orange* (*cwo*). CWO enters the nucleus and inhibits CLK:CYC activity by binding to E-box elements of clock genes (Kadener et al., 2007; Lim et al., 2007; Richier et al., 2008). CWO acts also indirectly promoting CLK:CYC-mediated transcription, by repressing the production of the CLK:CYC inhibitor CLOCK INTERACTING PROTEIN CIRCADIAN (CIPC; Rivas et al., 2021). *Cipc* transcription modulation might occur at the level of the E-boxes detected in the *Cipc* locus (red question mark; Rivas et al., 2021). Phosphorylation mediated by DOUBLETIME (DBT), CASEIN KINASE 2 (CK2), and SHAGGY (SGG) modulate clock protein activities, regulating protein–protein interactions, nuclear translocation, and degradation (Curtin et al., 1995; Saez and Young, 1996; Price et al., 1998; Rothenfluh et al., 2000; Martinek et al., 2001). Light activates the photoreceptor CRYPTOCHROME (CRY), which interacts with TIM and mediates its degradation in combination with JETLAG (JET). SUPERNUMERARY LIMBS (SLIMB) signals PER degradation (Emery et al., 1998; Stanewsky et al., 1998; Lin et al., 2001; Koh et al., 2006). **(B)** The first lepidopteran TTL includes CLK and BMAL1, which dimerize and bind to the E-boxes in the promoters of *per*, *tim*, and *cry2* genes. In the cytoplasm, PER, TIM, and CRY2 form a complex. CRY2, stabilized by PER, enters into the nucleus to inhibit CLK:BMAL1 activity (Zhu et al., 2005, 2008; Yuan et al., 2007). The second and third TTLs are speculative and include lepidopteran orthologs of *Drosophila* core and regulatory clock components identified in lepidopteran assembled genomes (Zhan et al., 2011; Derks et al., 2015). In the second TTL, the CLK:BMAL1 complex induces the transcription of *vri* and *Pdp1* genes, which, once translated, compete for the same element (VP-box) in the *Clk* promoter, controlling *Clk* transcription. In the third TTL, a CLK:BMAL1 dimer activates the transcription of *cwo*. CWO enters the nucleus and inhibits CLK:BMAL1 activity by binding to E-box elements of clock genes. Phosphorylation mediated by DBT, CK2, and SGG modulate clock protein activities, regulating protein–protein interactions, nuclear translocation, and degradation. Light is responsible for TIM degradation in a process mediated by CRY1 and JET. SLIMB is involved in PER degradation. Pathways triggering to CRY2 degradation are still unknown (black question mark). Dashed arrows indicate degradation pathways; sinusoidal lines show transcription activity.

The core of the molecular circadian clock is driven by positive and negative interlocking transcription–translation feedback loops (TTLs), which sustain a rhythmic production of mRNA and proteins of key clock factors (recently reviewed in Patke et al., 2020). In *D. melanogaster*, the positive limb of the major TTL includes the basic helix–loop–helix (bHLH), Per-Arnt-Sim (PAS), transcriptional factors dCLOCK (dCLK) and dCYCLE (dCYC, an ortholog of BMAL1), which acting as a heterodimer (dCLK:dCYC), bind conserved sequences (E-boxes) in the promoters of d*period* (d*per*) and d*timeless* (d*tim*), activating their transcription. dPER and dTIM proteins belong to the negative limb of the major TTL and act in the nucleus as inhibitors of the dCLK:dCYC transcriptional regulators (Allada et al., 1998; Bae et al., 1998; Darlington et al., 1998; Rutila et al., 1998; Glossop et al., 1999; Lee et al., 1999; Figure 2A). However, at both post-transcriptional and post-translational levels d*per* and d*tim* mRNAs and proteins are subjected to several modifications which control dPER and dTIM synthesis, stability, accumulation, and nuclear translocation, generating fundamental temporal delays in the TTL, and in turn determining the precise timing of the circadian clock (Curtin et al., 1995; Saez and Young, 1996; Price et al., 1998; Rothenfluh et al., 2000; Martinek et al., 2001; Patke et al., 2020). In a second TTL, dCLK:dCYC dimer induces the expression of dVRILLE (dVRI) and dPAR DOMAIN PROTEIN1ε (dPDP1ε), which inhibits and promotes d*Clk* expression, respectively (Cyran et al., 2003; Figure 2A). A third TTL helps sustain a robust rhythmic oscillation and includes the transcriptional repressor d*clockwork orange* (d*cwo*; Patke et al., 2020). d*cwo* transcription is promoted by dCLK:dCYC, and dCWO modulates dCLK:dCYC activity. In particular, dCWO represses dCLK:dCYC-mediated transcription by binding to E-box elements of clock genes, including d*per* and d*tim* (Kadener et al., 2007; Lim et al., 2007). Recent data indicate that CWO also exerts an indirect promotion of dCLK:dCYC activity, by repressing transcription of the *Drosophila* ortholog of mouse *Clock Interacting Protein Circadian* (*Cipc*), an inhibitor of dCLK:dCYC activity (Rivas et al., 2021; Figure 2A).

For virtually all organisms, light is the main environmental stimulus that entrains the circadian clock with the 24-h LD cycle. In *Drosophila*, light synchronization is mainly determined by the blue light photoreceptor dCRY (Emery et al., 1998; Stanewsky et al., 1998). Once activated by light, dCRY interacts with dTIM inducing its degradation which in turn impacts dPER stability, thereby resetting the molecular clock (Lin et al., 2001; Koh et al., 2006; Figure 2A).

The molecular elements among insect circadian clocks are homologous, although, some important differences have been identified between the circadian factors of *Drosophila* and Lepidoptera (Figure 2B). PER, TIM, CLOCK, and BMAL1 clock components from *A. pernyi* have been demonstrated to act in the positive (ApCLOCK, ApBMAL1) and negative (ApPER, ApTIM, the latter with a modulatory activity) limbs of the major TTL, using *Drosophila* embryonic Schneider (S2) cells (Chang et al., 2003). However, a bioinformatic analysis mapped the main transactivation domain essential for the ApCLK:ApBMAL1 function in the C-terminal region of ApBMAL1 protein, and not at the level of ApCLK, as previously shown for dCLK in *Drosophila* (Allada et al., 1998; Darlington et al., 1998; Chang et al., 2003). This appears a common feature in Lepidoptera, as the presence of a BMAL1 transactivation domain was reported in other species belonging to this order, including *B. mori* and *D. plexippus* (Markova et al., 2003; Zhang et al., 2017). Interestingly, with the exception of *Drosophila*, the localization of the transactivation domain in the BMAL1 C-terminal region is highly conserved in other non-lepidopteran insects and vertebrates (Chang et al., 2003). These observations suggested an evolutionary model, in which the common ancestor of insects and vertebrates possessed a BMAL homolog characterized by a C-terminal transactivation domain. The latter was subsequently lost in the *Drosophila* lineage, likely because of the acquisition of a functionally related transactivation domain within dCLK (Chang et al., 2003). For these reasons, in this review, we use “BMAL1” to identify the lepidopteran ortholog of *Drosophila* dCYC, although, both BMAL1 and CYC are currently employed in the lepidopteran circadian clock literature.

The most informative experiments elucidating the structure and functioning of the lepidopteran molecular clock have been performed in *D. plexippus*, in a huge series of studies initiated by Reppert and colleagues to understand the role of the circadian clock in the seasonal migration typical of this butterfly (Mouritsen and Frost, 2002; Froy et al., 2003; Zhu et al., 2005, 2008; Zhan et al., 2011; Table 1). These investigations demonstrated that *D. plexippus* possesses two CRY proteins, one ortholog to the light-sensitive *Drosophila* dCRY (henceforth DpCRY1) and a second vertebrate-like light-insensitive CRY, named DpCRY2 (Figure 2B). DpCRY1 was able to mediate the light-sensitive degradation of DpTIM in monarch butterfly DpN1 embryonic cell lines (Palomares et al., 2003; Zhu et al., 2008). On the other hand, DpCRY2 coimmunoprecipitated with DpPER and DpTIM in both DpN1 cells and *D. plexippus* brains and showed an inhibitory activity of the DpCLK:DpBMAL1-mediated transcription in *Drosophila* and monarch cell lines (Zhu et al., 2005, 2008; Yuan et al., 2007). In addition, DpCRY2 was the only *D. plexippus* clock factor showing circadian translocation into the nuclei of the putative *Danaus* brain clock neurons (see below), while the role of DpPER was associated with a DpCRY2 stabilization rather than with an active transcriptional repression (Zhu et al., 2008). As a consequence, a *Danaus* molecular clock model in which DpCRY2 represents the major inhibitor of the DpCLK:DpBMAL1 clock-controlled transcription in the first TTL was developed (Zhu et al., 2008; Zhan et al., 2011; Figure 2B). This model was further confirmed by the generation of *D. plexippus* Dp*cry2* and Dp*Clk* knock-out (KO) mutants, which indicated DpCRY2 and DpCLK, respectively, as a transcriptional repressor and a transcriptional activator of the monarch circadian clock *in vivo* (Merlin et al., 2013; Markert et al., 2016). In addition, the analysis of CRISPR/Cas9-mediated *D. plexippus* mutants with different modifications in the DpBMAL1 C-terminal region and luciferase assays in *Drosophila* cells mapped the amino acid portions of DpBMAL1 and DpCLK important for the DpCRY2 repression activity and regulation of circadian rhythmicity (Zhang et al., 2017). It was demonstrated that the DpCRY2 inhibitory activity on DpCLK:DpBMAL1 occurs with a dual mechanism. In particular, one mechanism which appears to be independent from the DpBMAL1 transactivation domain, involves a specific residue of DpCLK (E333 in the PAS-B domain) and it is important for the generation of circadian rhythmicity. The second mechanism implies the interaction with both the DpBMAL1 transactivation domain and DpCLK (at the residue W328) and plays a role in the regulation of the circadian phase or period (Zhang et al., 2017).

The *D. plexippus* molecular clockwork, with CRY2 acting as main transcriptional repressor of the CLK:BMAL1 dimer in the first TTL, is currently considered the model of the molecular circadian clock in Lepidoptera, as two functional *cry* genes have been detected in several species, including those belonging to the superfamilies of Bombycoidea (*B. mori* and *A. pernyi*; Zhu et al., 2005; Kawamoto et al., 2019), Noctuoidea (*S. littoralis*, *S. litura*, *A. segetum*, the pink stalk borer *Sesamia nonagrioides*, and the cotton bollworm *Helicoverpa armigera*; Merlin et al., 2007; Tomioka and Matsumoto, 2010; Yan et al., 2013; Chang et al., 2019; Zhang et al., 2021) and Geometroidea (the winter moth *Operophtera brumata*; Derks et al., 2015).

Finally, the orthologs of other *Drosophila* core and regulatory circadian clock components have been identified with the assembly of several Lepidoptera genomes, including those of *D. plexippus* and *O. brumata*, resulting in a lepidopteran molecular circadian clock model incorporating three main TTLs (Zhan et al., 2011; Derks et al., 2015; Figure 2B). Further work will be fundamental to functionally demonstrate the role of all these elements in the molecular clock of this order.

Currently, the molecular circadian clock has been deeply characterized in a relatively small number of Lepidoptera. Nevertheless, the knowledge derived from these studies has given a fundamental contribution to the comprehension of circadian clock evolution in insects. In particular, the identification of two functionally different *cry* genes in Lepidoptera led to the detection of both *cry1* and *cry2* in other dipteran non-Drosophilid species such as the mosquitoes *Anopheles gambiae* and *Aedes aegypti* (Zhu et al., 2005; Yuan et al., 2007). On the contrary, species such as the hymenopteran *Apis mellifera* and *Bombus impatiens* as well as the coleopteran *Tribolium castaneum* have been demonstrated to possess only *cry2* (Rubin et al., 2006; Yuan et al., 2007). When tested in *Drosophila* cell cultures, CRY2s belonging to these species were not light-sensitive and behaved as repressors of a CLK:BMAL1-mediated transcription (Yuan et al., 2007). A clock bearing only light-insensitive *cry2* has also been detected in the fire ant *Solenopsis invicta*, belonging to the order Hymenoptera (Ingram et al., 2012). These findings led to hypothesize that the Lepidoptera-like molecular clock, with both a light-sensitive CRY1 and a transcriptional repressor CRY2, represents an ancestral clock, while those characterized by only a functional CRY1 (as in *Drosophila*) or only a functional CRY2 (as in bees, beetles, and ants) are derived clocks, in which *cry2* or *cry1* have been lost, respectively (Reppert, 2007; Yuan et al., 2007; Merlin and Reppert, 2009).

### The Circadian System Organization at the Organismal Level

#### The Brain Central Clock in Adult Lepidoptera

In Lepidoptera, the central clock controlling the rhythmicity of several behavioral phenotypes resides in the brain. This was first demonstrated in *A. pernyi* and *H. cecropia* by transplantation and ablation experiments, which showed that the dorsolateral brain region houses a master clock controlling circadian behavioral phenotypes such as adult eclosion and flight (Truman, 1972, 1974).

**Figure 3:**
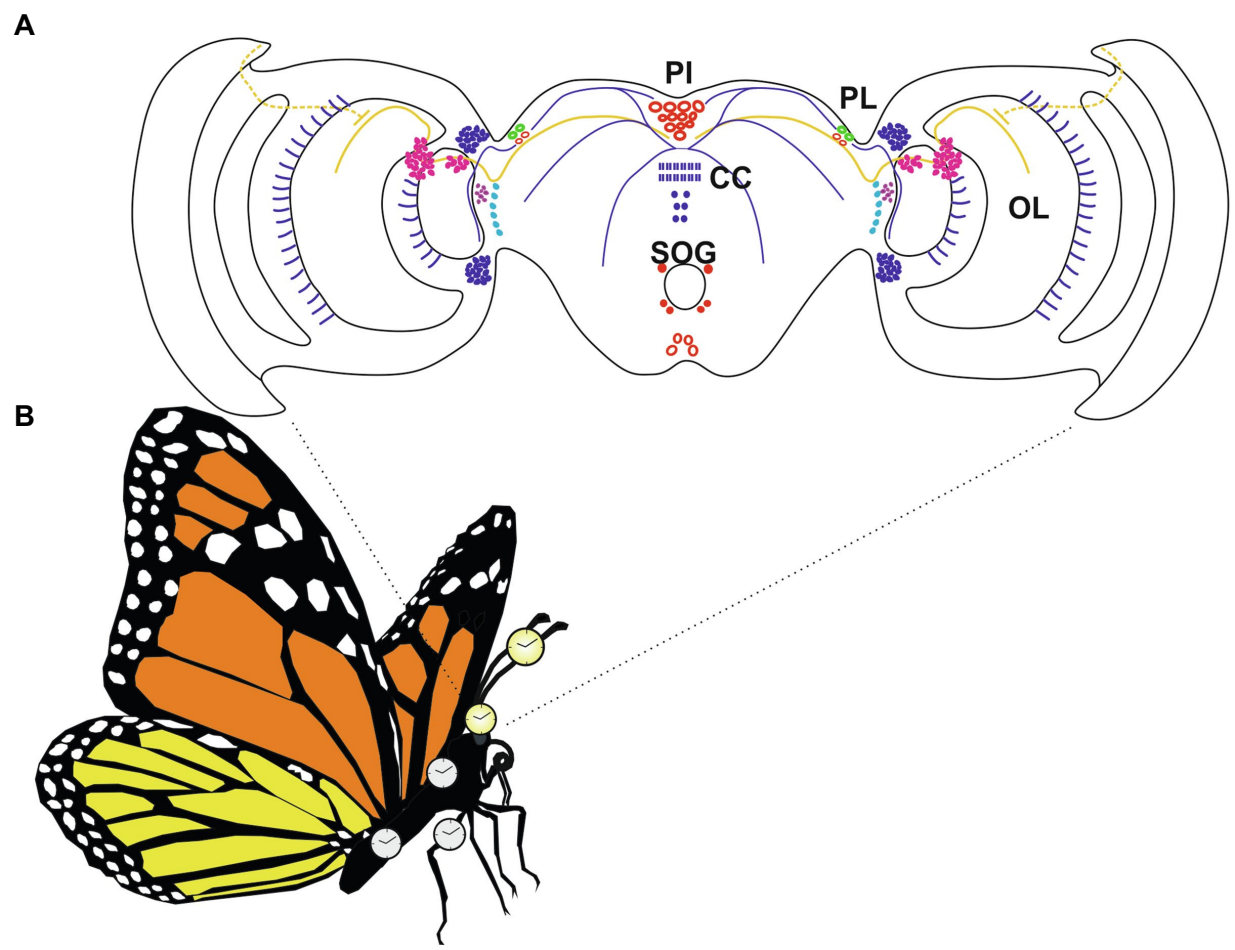
The circadian system in *Danaus plexippus* adults, with multiple oscillators located in the brain and body. **(A)** Relative positions of neurons and fibers expressing clock factors in *D. plexippus* brain. In each brain hemisphere, two Pars Lateralis (PL; green) neurons co-express DpTIM, DpPER, DpCRY1, and DpCRY2. These PL cells are believed the putative circadian clock neurons of the *D. plexippus* brain. Neurons expressing DpTIM, DpPER, DpCRY1, and/or DpCRY2 are depicted in red. In these cells, the four clock molecules partially colocalize. Regions expressing DpTIM or DpCRY1 are indicated in magenta. In these areas, TIM and CRY1 do not colocalize. Cells exclusively expressing TIM or PER are represented in purple and light blue, respectively. CRY2-positive neurons and fibers are shown in blue. Continuous yellow lines indicate fibers expressing CRY1. Dotted yellow lines indicate projections of the dorsal rim area. PL, Pars lateralis; PI, Pars intercerebralis; CC, central body; SOG, Subesophageal ganglion; and OL, optic lobe (modified from Sandrelli et al., 2008; see text for explanation). **(B)** Schematic representation of an adult monarch butterfly. Yellow clocks represented autonomous oscillators located in the brain and antennae (Sauman et al., 2005; Zhu et al., 2008; Merlin et al., 2009). White clocks indicate monarch butterfly tissues (thorax, legs, and abdomen) which showed a rhythmic expression of different clock factors in LD conditions (Merlin et al., 2009) and are likely sites of peripheral oscillators.

Most of the studies aimed at identifying the brain master clock neurons in Lepidoptera have been performed in four species: *A. pernyi* (Sauman and Reppert, 1996; Mohamed et al., 2014), *M. sexta* (Wise et al., 2002), *B. mori* (Sehadová et al., 2004), and *D. plexippus* (Sauman et al., 2005; Zhu et al., 2008). To map the clock neurons within the brains of these organisms, *in situ* mRNA hybridization analyses and/or immunolocalization studies using specific antibodies against different clock proteins have been performed. Several species-specific differences in brain expression patterns have been detected, with *M. sexta* and *A. pernyi* showing the highest and the lowest number of clock gene expressing neurons, respectively (Sauman and Reppert, 1996; Wise et al., 2002; Mohamed et al., 2014). However, a common feature characterizing the four species was the expression of the analyzed clock factors in two clusters of large type Ia_1_ neurosecretory cells, symmetrically located in each Pars Lateralis (PL) of the brain, which are considered to represent the circadian clock neurons in the brain of these Lepidoptera (Wise et al., 2002; Sehadová et al., 2004; Sauman et al., 2005; Zhu et al., 2008; Figure 3A).

In the *A pernyi* central brain, four PL neurons per hemisphere have been reported as the only cells expressing ApPER and ApTIM (Sauman and Reppert, 1996). More recently, ApPER-/ApTIM-positive PL neurons have been shown to co-express ApBMAL1 and ApCLK proteins (Mohamed et al., 2014). In LD conditions, ApPER and ApTIM were rhythmically expressed in both cytoplasm and axonal projections, with higher levels during the night and lower during the day. However, the expression profiles of ApPER and ApTIM proteins oscillated with the same phase of Ap*per* and Ap*tim* mRNAs, without any delay between mRNA and protein accumulation (Sauman and Reppert, 1996). In addition, the staining was restricted to cytoplasmic and axonal subcellular compartments, while no ApPER and/or ApTIM signals were detected within the nuclei (Sauman and Reppert, 1996). These data provided the first indication that the functioning of these elements in the putative lepidopteran adult clock neurons could be different from that of *Drosophila*, as later shown in the monarch butterfly (Sauman et al., 2005; Zhu et al., 2008).

In *M. sexta*, only the distribution of Ms*per* mRNA and its corresponding protein was analyzed in the adult brain (Wise et al., 2002). In PL neurons, MsPER protein was localized in both the cytoplasm and nucleus, but no evident 24h cycling expression was detected (Wise et al., 2002).

In *B. mori* adult brains, PL neurons expressed different clock factors, including BmPER, BmCYC, and BmCRY-like proteins. At this level, the staining of all the proteins was cytoplasmatic, with only BmPER signal showing a cycling intensity in LD (Sehadová et al., 2004).

In *D. plexippus*, the four PL neurons of each brain hemisphere expressed DpTIM and DpCRY2. In each PL cluster, two neurosecretory cells also co-expressed DpPER and DpCRY1, and are considered the circadian clock neurons in the monarch brain (Sauman et al., 2005; Zhu et al., 2008; Figure 3A). In these neurons DpTIM and DpPER showed a cycling expression in both LD and DD, with a peak during the night. However, DpTIM, DpPER, and DpCRY1 were localized in the cytoplasm. On the contrary, DpCRY2 showed a cycling expression and a localization at the nuclear level at the beginning of the day, when the maximal transcriptional repression of Dp*per* mRNA is recorded in the monarch brain (Zhu et al., 2008).

In all these species, apart from *A. pernyi*, additional expression of the examined clock factors have been reported in other regions of the brain, including the Pars Intercerebralis (PI), a region known to have a role in insect insulin signaling, aging and diapause, as well as the optic lobe (OL), and subesophageal ganglion (SOG; Wise et al., 2002; Sehadová et al., 2004; Sauman et al., 2005; Zhu et al., 2008). In *B. mori*, PI neurons expressing BmPER appeared at the identical position to those expressing BmCYC (Sehadová et al., 2004). In the monarch butterfly, several PI neurons expressed DpTIM, DpPER, DpCRY1, and DpCRY2, and a cluster of neurons clearly co-expressed DpTIM, DpCRY1, and DpPER (Sauman et al., 2005; Zhu et al., 2008; Figure 3A). Moreover, both DpCRY1 and DpCRY2 have been detected in multiple neuronal projections connecting PL neurons to brain regions important for migration (Figure 3A). DpCRY1-positive fibers appeared to connect PL neurons to the dorsal rim area (DRA), an eye region involved in the perception of the polarized light input (Sauman et al., 2005; see below). DpCRY2-positive projections appeared to link PL and PI neurons, and cycling DpCRY2 expression was found in the fibers of the central complex, a conserved high-order neuronal structure of the insect brain, which in the monarch is considered the central site of the sun-compass (Zhu et al., 2008; reviewed in Reppert et al., 2016; Reppert and de Roode, 2018). Interestingly, a recent analysis mapped the brain expression of *per* and *tim* genes in *E. kuehniella* (superfamily Pyraloidea), a pest moth which in the evolution of Lepidoptera represents a less divergent species compared to *A. pernyi*, *B. mori*, and *M. sexta*, which belong to the Macroheterocera clade (Kobelková et al., 2015; Mitter et al., 2017; Figure 1). Immunolocalization studies detected a wide expression of EkPER throughout *E. kuehniella* brain, with hundreds of EkPER-positive neurons (Kobelková et al., 2015). In most of the *E. kuehniella* neurons, intense but non-cycling EkPER signals were detected at the level of both cytoplasm and nuclei. However, in the PI a group of 4–5 large neurosecretory cells showed EkPER localization restricted to the cytoplasm and neuronal projections, with only the latter displaying a daily rhythm in staining intensity, and higher signals during the night in LD conditions (Kobelková et al., 2015). To date, the localization of the master clock neurons in this species is still unknown. However, the high number of EkPER-positive neurons in its brain has been hypothesized to be a characteristic of an ancient clock, as already proposed for more ancestral Apterygota and Exopterygota, compared to holometabolous insects (Závodská et al., 2003; Kobelková et al., 2015).

#### Peripheral Clocks in Adult Lepidoptera

The presence of circadian clocks outside the brain has been demonstrated in adults of different lepidopteran species, including *A. pernyi*, *M. sexta*, *B. mori*, *S. littoralis*, *D. plexippus*, *E. kuehniella*, and *H. armigera* (Sauman and Reppert, 1996; Wise et al., 2002; Syrova et al., 2003; Iwai et al., 2006; Schuckel et al., 2007; Kotwica et al., 2009; Merlin et al., 2009; Kobelková et al., 2015; Yan et al., 2019). In insects, these circadian clocks appear to be important for the daily regulation of the physiological activities typical of peripheral tissues or organs (Tomioka et al., 2012). Some of these peripheral clocks depend on the clock located in the brain, others are self-sustained oscillators, autonomously ticking with respect to the master clock, as nicely demonstrated in the dipteran *D. melanogaster* (Plautz et al., 1997). Throughout expression studies and/or physiological analyses, peripheral clocks have been detected in different adult structures, such as eyes, antennae, thorax, abdomen, flight muscles, midgut, testis, and legs (Sauman and Reppert, 1996; Wise et al., 2002; Iwai et al., 2006; Schuckel et al., 2007; Merlin et al., 2009; Yan et al., 2014, 2019; Kobelková et al., 2015; Figure 3B). Here, we will focus on peripheral clocks located in the eye, antenna, and testis, for which most of the data are available.

Like all insects, Lepidoptera possess a compound eye, composed of units known as ommatidia, each formed by a variable number of photoreceptor cells (e.g., nine in Papilionoidea and Sphingidae; eight in Noctuoidea; Friedrich et al., 2006). Most of the studies aimed at characterizing lepidopteran eye clocks were conducted by mapping *per* mRNA and/or protein expression in *A. pernyi*, *M. sexta*, and *E. kuehniella* (Sauman and Reppert, 1996; Wise et al., 2002; Kobelková et al., 2015). In *M. sexta*, both Ms*per* mRNA and MsPER protein were detected in photoreceptor nuclei without any clear rhythmic expression (Wise et al., 2002). Conversely, in *A. pernyi* and *E. kuehniella* a rhythmic *per* mRNA accumulation was observed in the nuclei of these cells, with a maximum signal in the dark phase of the LD cycle. Moreover, several hours following the mRNA peak, intense PER protein staining was detected in the photoreceptor nuclei (Sauman and Reppert, 1996; Kobelková et al., 2015). Both the temporal delay between mRNA and protein accumulation and protein nuclear translocation are considered among the principal characteristics of the *Drosophila* circadian clock. However, they have not been reported for *per* in the *A. pernyi* and *E. kuehniella* brains (see above). These observations suggest that in *A. pernyi* and *E. kuehniella* the molecular mechanisms governing the circadian clock in the eye and central brain are distinct. This is not surprising since dissimilarities between the central and peripheral clocks have already been reported in *D. melanogaster*. In particular, dCRY which is fundamental for light synchronization in both fly brain and peripheral clocks, in antennae and Malpighian tubules has an additional role for maintaining a persistent oscillation, possibly acting as a transcriptional repressor in the core molecular clock (Ivanchenko et al., 2001; Krishnan et al., 2001; Tomioka et al., 2012). Finally, in the photoreceptors of the D. *plexippus* compound eye, only the expression of Dp*cry1* mRNA and its corresponding protein has been reported to date (Wan et al., 2021).

The compound eye perceives light cues and forms visual images (Sondhi et al., 2021), playing a key role for several insect behaviors, such as flight, foraging, mating, and predator avoidance. Additionally, within the compound eye, the dorsal rim area (DRA) is involved in detecting polarized light (Labhart and Meyer, 1999; Reppert et al., 2004). In the study of *D. plexippus* migration, sun and polarized light detected by the main retina and DRA of the compound eye have been demonstrated to represent the sensing information transmitted to the sun compass, that is likely located in the central complex of the brain (Froy et al., 2003; Reppert et al., 2004; Sauman et al., 2005; Heinze and Reppert, 2011, 2012).

At a molecular level, light is perceived by opsins, G-coupled seven-transmembrane proteins, which combined with a chromophore are key light-sensing factors for color vision. In Lepidoptera and other insects, five distinct opsins have been detected in both nocturnal and diurnal species (Feuda et al., 2016). Among these, the most studied are the long wavelength sensitive opsin (peak absorbance 500–600nm), the blue sensitive opsin (peak absorbance 400–500nm) and the UV sensitive opsin (peak absorbance 300–400nm; Sondhi et al., 2021). mRNA *in situ* hybridization studies in the monarch showed that the three opsins are expressed in the compound eye, except for the DRA in which only the UV opsin mRNA was detected (Sauman et al., 2005).

A quantitative PCR study on *H. armigera* compound eyes showed a daily rhythmic oscillation in the transcription levels of the three opsins, with a peak of expression during daytime, in LD conditions and the first day of DD (Yan et al., 2014). However, the daily mRNA oscillations dumped from the second day of DD and in constant light (LL), indicating an interaction between the circadian clock and environmental light in the control of opsin expression in this organism (Yan et al., 2014). The molecular mechanism connecting opsin daily expression and circadian clocks is still unknown. However, cycling mRNA levels of different core clock genes (Ha*per*, Ha*tim*, Ha*cry1*, and Ha*cry2*) have been detected in *H. armigera* eyes, suggesting the presence of a peripheral clock (Yan et al., 2014, 2019).

A better described clock is located in the antennae, appendages of the adult lepidopteran head involved in detecting odor, taste, temperature, humidity variations, and mechanoreception. Antennae are long and slight structures, composed of two basal segments and a flagellum, formed of multiple units (Hansson and Stensmyr, 2011). Antennae play a fundamental role in reproduction as they are involved in pheromone detection, which occurs in a specific time window of the day and is under circadian control in several species (Groot, 2014; Table 1).

The presence of a peripheral clock in the lepidopteran antenna has been suggested by electrophysiological studies demonstrating in *M. sexta* and *S. littoralis* that the electroantennogram (EAG) response after olfactory stimuli shows a daily variation in both LD and DD conditions (Hoballah et al., 2005; Merlin et al., 2007; Fenske et al., 2018). Additionally, cycling mRNA and/or protein expression of different circadian clock factors was detected in the antennae of several lepidopteran species, including *B. mori* (Bm*per*; Iwai et al., 2006), *M. sexta* (Ms*per*; Schuckel et al., 2007), and *D. plexippus* (Dp*per*, Dp*tim*, and Dp*cry2*; Merlin et al., 2009). Importantly, in the monarch the daily oscillation in DpTIM was maintained in explanted antennae, cultured in both LD and DD conditions (Merlin et al., 2009). Moreover, DpTIM abundance was consistently lower during the light phase of the day compared to the night, independently from the phase of the 24h LD cycle, indicating that the *D. plexippus* antenna possesses an autonomous light-entrainable circadian clock (Merlin et al., 2009; Figure 3B).

In the monarch butterfly, the antennal peripheral clock has been implicated in the circadian compensation of the sun compass, as migratory *D. plexippus* with an intact brain but without any functional antennae lost the time-compensated feature of the sun compass when analyzed in a flight simulator (Merlin et al., 2009; Guerra et al., 2012). However, butterflies with a single functional antenna showed an operating time-compensated sun compass, indicating that each antennal structure can provide the timing information to the sun compass brain center, in a neuronal circuit that has still to be fully characterized (Guerra et al., 2012).

A peripheral circadian clock controlling the timing of sperm release from the male testis has been suggested by studies performed in *C. pomonella* (superfamily Tortricoidea; Giebultowicz and Brooks, 1998), as well as in the gypsy moth *Lymantria dispar* and *S. littoralis* (superfamily Noctuoidea; Giebultowicz et al., 1997; Syrova et al., 2003). Expression analyses have indicated a rhythmic expression of *per* mRNA and/or the corresponding protein at a testis level in *C. pomonella*, *B. mori*, and *S. littoralis* (Gvakharia et al., 2000; Iwai et al., 2006; Kotwica et al., 2009). Additionally, in a *S. littoralis* functional study, an *in vitro* Sp*per* RNA interference (RNAi) caused a delay in sperm release, indicating that a molecular oscillator involving Sp*per* plays an essential role in regulating rhythmic sperm release in this species (Kotwica et al., 2009).

#### The Circadian Clock During Development

Several studies aimed to characterize the molecular circadian clock during development in different lepidopteran species. Although in most of the cases, such investigations are at an early stage, compelling evidence indicates that their results are both important for understanding the regulation of the different circadian rhythms occurring in embryonic and larval stages and can provide useful information for the management of both pest and beneficial Lepidoptera, as discussed below.

A circadian clock driving egg-hatching rhythmicity has been shown in several species from mid-embryogenesis (Table 1). *Antheraea pernyi* transplantation experiments demonstrated that the circadian clock controlling egg-hatching rhythm resides in the brain (Sauman and Reppert, 1998). Moreover, a fundamental role of TIM and PER in this circadian output has been recently confirmed in *B. mori* Bm*tim* and Bm*per* KO mutants (Ikeda et al., 2019; Nartey et al., 2020). In particular, Bm*tim* KO egg-hatching activity was significantly different compared to that of the wild-type, when monitored every 4h in 12:12 LD and DD conditions, suggesting an egg-hatching rhythm alteration in Bm*tim* KO individuals (Nartey et al., 2020). Additionally, an accurate analysis was performed on Bm*per* KO mutants, recording hatching every 30min, in both 12:12 LD and DD regimes (Ikeda et al., 2019). In DD conditions, Bm*per* KO mutants lost egg-hatching rhythm, indicating a disruption of the circadian clock (Ikeda et al., 2019). In 12:12 LD, Bm*per* KO animals displayed a main hatching peak 1h after light-on, likely produced by a direct response to light. However, differently from controls, these mutants showed additional hatching activities also at other times of the 24hday (Ikeda et al., 2019). These data were also consistent with the wider “egg-hatching gate” characterizing Bm*per* knockdown (KD) *B. mori* lines when compared to controls under LD regimes, a result indicating that a Bm*per* KD moderately affected egg-hatching rhythms (Sandrelli et al., 2007).

The presence of a maturing circadian clock in the brain of developing embryos was first shown in *A. pernyi*, in which ApTIM and ApPER proteins were found in the cytoplasm of the PL neurons in 4- and 6-day-old embryos, respectively (Sauman et al., 1996). In these neurons, ApTIM and ApPER signals did not show any cycling variation or nuclear localization; however, during embryonic maturation and in the first instar larvae initially ApTIM and, subsequently, ApPER were also observed in the axon projections of PL neurons (Sauman et al., 1996).

More recently, a cycling expression of several clock genes, including Bm*per*, Bm*tim*, Bm*cry1*, Bm*cry2*, and *BmClk* was demonstrated in *B. mori* embryos under both LD and DD conditions (Xu et al., 2011; Tao et al., 2017; Ikeda et al., 2019; Nartey et al., 2020). Additionally, immunolocalization studies showed BmPER nuclear translocation after a transient light pulse in late-stage embryonic heads, suggesting that a molecular feedback loop including Bm*per* controls egg-hatching rhythm in *B. mori* (Tao et al., 2017). Interestingly, in Bm*per* KO embryos the levels of Bm*per* mRNAs did not vary during the day and were extremely low when compared to those of wild-type individuals (Ikeda et al., 2019). As the lack of an operating PER/TIM-negative feedback loop would predict constitutively higher Bm*per* expression levels, Ikeda et al. (2019) hypothesized that a nonsense-mediated mRNA decay (NMD) phenomenon affecting the stability of the aberrant Bm*per* mRNA was active in Bm*per* KO mutants. NMD is a protecting mechanism acting as mRNA surveillance, which induces the degradation of mRNAs carrying non-sense mutations in all eukaryotes, including *B. mori* (Houseley and Tollervey, 2009; Komoto et al., 2009). NMD might also explain the low Bm*tim* expression levels detected in Bm*tim* KO embryos in both LD and DD regimes (Nartey et al., 2020).

Besides the brain, other tissues express clock factors during embryonic development. In *A. pernyi*, for example, non-cycling ApPER staining was detected in the cytoplasm of fat body cells (Sauman et al., 1996). Additionally, in the midgut epithelium of *A. pernyi* embryos and first instar larvae, ApPER showed a cycling profile in LD and DD and a rhythmic nuclear translocation occurring late in the night in LD conditions (Sauman et al., 1996; Sauman and Reppert, 1998). However, head ligation experiments in first instar larvae disrupted ApPER nuclear translocation demonstrating that the gut peripheral clock depends on the brain master clock (Sauman and Reppert, 1998). Although extensive and standardized studies have not always been performed, several tissues have been shown to express multiple clock factors during larval development in species such as *B. mori*, *S. litura*, and *S. littoralis*. Specifically, last (5th) instar larvae of *B. mori* actively transcribe the clock genes Bm*tim*, Bm*per*, Bm*Clk*, and Bm*Bmal1* in the head, midgut, fat body, and silk glands (Markova et al., 2003; Iwai et al., 2006; Xu et al., 2011; Nobata et al., 2012). In the midgut, a significant cycling expression of Bm*tim* was demonstrated in both LD and DD, while Bm*per* mRNA levels tended to be higher during the night compared to the day (Nobata et al., 2012). Among the multiple cell types composing the silkworm midgut, BmPER staining was limited to goblet cells, known for their role in the active transport of potassium from hemolymph into the gut lumen and in the release of digestive enzymes (Daimon et al., 2008; Park et al., 2014). Future work employing the recently generated Bm*per* KO mutants (Ikeda et al., 2019) might functionally clarify the meaning of the goblet cell-restricted BmPER expression in the silkworm midgut.

Transcriptomic and quantitative PCR experiments performed on the head, midgut, and fat body of *S. litura* larvae at the last (6th) developmental stage showed a daily cycling expression in several clock genes in LD conditions (Slitu*per*, Slitu*tim*, Slitu*cry1*, Slitu*cry2*, Slitu*Cwo*, and Slitu*vri* with unimodal expression profiles; Slitu*Bmal1* and Slitu*Clk* displaying bimodal expression profiles; Zhang et al., 2021). To date, only *S. litura* larval heads have been analyzed in DD conditions, showing a rhythmic expression of Slitu*Cwo*, Slitu*Bmal1*, and Slitu*Clk* mRNAs, while Slitu*per* and Slitu*tim* mRNA levels did not display any cycling profile (Zhang et al., 2021). Insect heads are composed of different structures, and a detailed clock gene expression map of the larval brain is still missing. However, these data indicate that a fully mature circadian clock bearing a self-sustained oscillation of *per* and *tim* components is absent in *S. litura* larvae. Nevertheless, it has been suggested that other molecular clock mechanisms, independent from Slitu*per* and/or Slitu*tim* mRNA oscillation(s), can guarantee an endogenous timekeeping system at this developmental stage, since *S. litura* larvae show circadian rhythmicity in feeding, defecation, and locomotor behaviors from the 5th instar onward, in both LD and DD conditions (Zhang et al., 2021; Table 1). It will be interesting to evaluate whether these behavioral rhythms are in some ways associated with circadian variations occurring at a protein level, including activity, post transcriptional modification/s, and nuclear translocation of the different elements composing the larval molecular clock.

Similar expression studies have been conducted in *S. littoralis*, a closely related species to *S. litura*, showing a cycling expression in different circadian genes in both midgut and fat body of last instar larvae, in LD and DD conditions (midgut: Sp*tim*; Sp*cry1*, and Sp*cry2*; fat body: Sp*per*; Sp*tim*, Sp*cry2*, and Sp*vri*; Suszczynska et al., 2017). However, the absence of a Sp*per* mRNA cycling transcription in DD at the midgut level prompted authors to suggest that this organ lacks a canonical molecular circadian clock. On the contrary, in fat bodies Sp*per* and Sp*tim* rhythmic expressions persisted in DD and a rhythmic SpPER protein nuclear translocation occurred in both LD and DD, suggesting that a mature circadian clock is operating in fat body of *S. littoralis* larvae (Suszczynska et al., 2017).

## The Role of the Circadian Clock in Lepidopteran Feeding Behaviors

In nature, adult moths and butterflies are crucial pollinators feeding on nectar, and in some cases, on pollen. However, almost all lepidopteran larvae are voraciously phytophagous, feeding on leaves and, less frequently, on stems, flowers, fruits, seeds, or wood. Complex plant-Lepidoptera interactions have evolved, with plants having developed defense strategies to protect from caterpillar attacks (Howe and Jander, 2008), while also attracting adult lepidopteran pollinators (Raguso, 2008; Rausher, 2008). Conversely, caterpillars have developed mechanisms to avoid plant defense systems (e.g., expansion of detoxification genes; Cheng et al., 2017; Gouin et al., 2017; Pearce et al., 2017) and adult moths and butterflies have evolved structures to efficiently reach their food sources, working at a same time as pollinators (Powell et al., 1998; Mitter et al., 2017; Kawahara et al., 2019; Figure 1). In this survival game, circadian systems of both plants and Lepidoptera appear to have played a fundamental role in controlling the timing of different activities/physiological phenomena, such as the emission (plants)/perception (moths/butterflies) of attraction signals, or the production of toxic substances (plants) and detoxification enzyme (caterpillars) at the proper time of the day.

**Figure 4:**
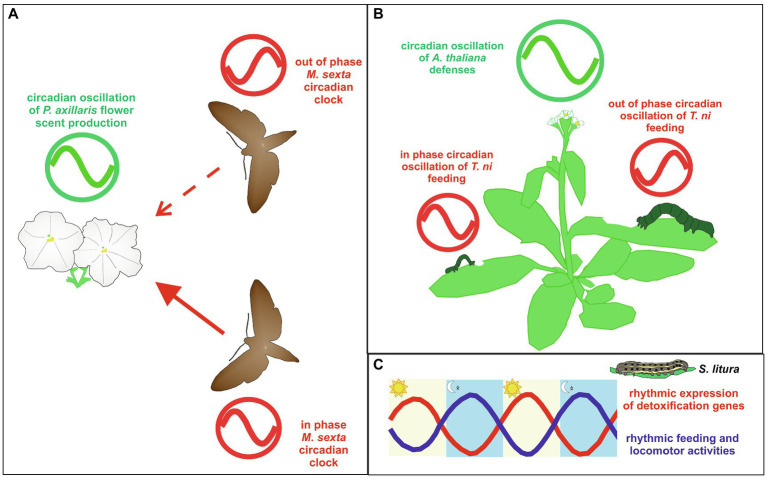
Examples of circadian clock-controlled interactions between Lepidoptera and plants. **(A)** The interplay between *Manduca sexta* and *Petunia axillaris* circadian clocks. *Petunia axillaris* flower scent production displays a circadian oscillation with a peak during the night (green clock). When *M. sexta* and *P. axillaris* clocks are synchronized (in phase), moths show higher levels of flower visits regarding moths with a circadian clock shifted by 12h compared to the plant clock (out of phase; Fenske et al., 2018). *Manduca sexta* circadian clocks are indicated in red. Solid and dashed red lines represent the higher and lower number of flower visits, respectively. **(B)** *Trichoplusia ni* caterpillars feeding on *Arabidopsis thaliana*. *Arabidopsis thaliana* jasmonate-mediated defenses show a circadian oscillation, which peaks during the day (green clock). When *T. ni* and *A. thaliana* clocks are synchronized (in phase), caterpillars feed less and, in turn, grow less rapidly compared to caterpillars with an internal clock shifted by 12h compared to the plant clock (out of phase; Goodspeed et al., 2012). *Trichoplusia ni* circadian clocks are indicated in red. **(C)** Schematic representations of the daily oscillations in locomotor and feeding activities (blue line) and expression of detoxification genes (red line) in *Spodoptera litura* caterpillars. In light/dark (LD) conditions, midguts and fat bodies show a 24h rhythmic transcription of detoxification genes with a peak during the daytime, when larvae are inactive, do not feed, and show a digestion activity at the highest levels (Zhang et al., 2021; see text for explanation).

A series of studies aimed at exploring the relationship between *M. sexta* moth and the plant *Petunia axillaris* indicate that an interaction between their respective circadian-controlled phenotypes is ecologically and evolutionary important for an efficient mutualistic relationship in which a plant requiring out-crossing provides nectar as a source of food to adult Lepidoptera which act as pollinators (Hoballah et al., 2005; Broadhead et al., 2017; Fenske et al., 2018; Figure 4A). Although, the *M. sexta* larval form is a pest for *Solanaceae* crops, adult moths are generally known as crepuscular/nocturnal nectivores of different plants species, including *P. axillaris* (Metcalf et al., 1993; Hoballah et al., 2005; Riffell and Hildebrand, 2016). *Petunia axillaris* flowers show clear rhythmicity in scent emission, with a peak during the night, in a mechanism under circadian control (Hoballah et al., 2005; Fenske and Imaizumi, 2016). *Manduca sexta* shows circadian rhythmicity in flight behavior, with higher activity during the night in both LD and DD (Broadhead et al., 2017; Fenske et al., 2018). Manipulating the circadian system of both moths and plants, Fenske et al. (2018) demonstrated that the moth and petunia circadian clocks must be in phase to ensure an efficient level of flower visits by the moths (Figure 4A). In fact, when the moth clocks were out of phase compared to that of the plant and vice versa, the number of flower visits was reduced compared to a condition in which the plant and moth circadian systems oscillated in phase. The importance of the circadian clock was emphasized by the fact that moth flight activity increased significantly when exposed to flower scent during the subjective night compared to when the same odor signal was administered during the subjective day. It has been demonstrated that the ability to perceive odors resides in the antenna, which also contains a peripheral clock in *M. sexta* moths (Schuckel et al., 2007). Additionally, in response to synthetic flower scent, *M. sexta* antennae show circadian rhythmicity in electrophysiological activity, with a peak during the night when the moths display greater foraging behavior (Hoballah et al., 2005; Fenske et al., 2018). Therefore, it can be hypothesized that in the moth the central and the peripheral clocks interact to coordinate the organismal locomotor and odor perception circadian activities.

In the study of the interactions between plants and Lepidoptera there is also evidence indicating that an in phase synchronization between the two organismal circadian clocks have likely promoted a clock-controlled mechanism enhancing the plants resistance to caterpillar diurnal attacks. One such example derived from a study analyzing the relationship between the circadian clocks of the generalist herbivore *T. ni* and the model plant *Arabidopsis thaliana* (Goodspeed et al., 2012; Figure 4B). *Trichoplusia ni* is commonly known as the cabbage looper since larvae preferentially feed on crucifers but successfully feed on over 160 plant hosts. *Arabidopsis thaliana* possesses a well-characterized circadian system (Barak et al., 2000; Robertson, 2000), and can host cabbage looper larvae. When fed on *A. thaliana*, *T. ni* larvae showed a circadian feeding behavior, with a higher feeding activity during the end of the day and lower during the night, in both LD and DD conditions (Goodspeed et al., 2012; Table 1). However, it was demonstrated that *T. ni* growth was enhanced when the larval feeding rhythm was entrained to free-run out of phase with respect to *A. thaliana* circadian rhythmicity, and reduced when the caterpillar free-running rhythm was in phase with that of *A. thaliana*’s clock (Goodspeed et al., 2012; Figure 4B). The variable larval growth rate was linked to the interaction between the clock-controlled larval feeding behavior and the plant clock-mediated production of jasmonic acid, a hormone triggering plant herbivore defense (Reymond et al., 2000; Jander et al., 2001; Kessler and Baldwin, 2002; Howe and Jander, 2008), which shows a peak of accumulation in the middle of the day (Goodspeed et al., 2012). In fact, when caterpillars were fed on arrhythmic or jasmonate-defective *A. thaliana* mutants, their weight increment was independent of the phase of circadian feeding behavior (Goodspeed et al., 2012). These data suggest a protective effect of the circadian system in *A. thaliana*, as the circadian clock controlled phase patterns of jasmonate accumulation are consistent with an anticipated preparation to the peak of the circadian caterpillar feeding behavior, occurring at the end of the day (Goodspeed et al., 2012).

On the other hand, different works have indicated that the lepidopteran counterparts might have evolved circadian-controlled strategies for avoiding plant defense mechanisms. For example, the absence of any rhythmic daily profile in feeding behavior detected in the tobacco hornworm *M. sexta* when reared on an artificial diet has been suggested as a strategy to limit the negative effects of the diurnal defense mechanisms typical of its natural host *Nicotiana attenuata* (Reynolds et al., 1986; Kim et al., 2011; Jander, 2012). However, further experiments are required, since in some species, the rhythmicity in larval feeding behavior can be influenced by several environmental factors, including the type of diet. This is the case of the tobacco budworm *Heliothis virescens* (superfamily Noctuoidea), whose feeding behavior was arrhythmic when maintained on an artificial diet in LD laboratory conditions but rhythmic when reared on a corn-based diet in a greenhouse, with a LD and temperature cycling environment (Niepoth et al., 2018).

A large body of evidence indicating the presence of circadian-controlled mechanisms for escaping plant defense strategies derived from a series of studies performed on nocturnal *Spodoptera* spp., pest insects with a wide host range, including several plant species of economic importance. In particular, *S. exigua*, *S. littoralis*, and *S. litura* have shown rhythmic feeding behavior, with a peak at night, when maintained on artificial diet in LD conditions (Kim and Hong, 2015; Suszczynska et al., 2017; Zhang et al., 2021). In *S. littoralis* and *S. litura* this rhythmicity was shown to persist in DD, suggesting that feeding behavior is a true circadian rhythm in the larvae of both species (Suszczynska et al., 2017; Zhang et al., 2021; Table 1). *Spodoptera litura* larvae showed a similar circadian profile in their locomotor behavior, with a peak of activity during the night in both LD and DD conditions (Zhang et al., 2021; Table 1; Figure 4C). Interestingly, transcriptomic analyses on *S. litura* larval midguts and fat bodies in LD conditions showed daily rhythmicity in the transcriptional levels of detoxification genes with a peak during the daytime, when larvae were most inactive and their digestion activity was at its highest levels (Zhang et al., 2021; Figure 4C). Although, the same analyses have not been performed in DD conditions, the importance of the circadian clock in the control of detoxification processes was suggested by transient KD experiments indicating that different core clock elements (Slitu*Clk*, Slitu*Bmal1*, and Slitu*Cwo*) are involved in modulating the transcription of several detoxification genes, with SlituCLK and SlituBMAL1 acting as promoting factors and SlituCWO as an inhibitor (Zhang et al., 2021). A molecular link between the *S. litura* circadian system and detoxification rhythm was further suggested by *in vitro* luciferase assays in *S. litura* cell lines, indicating that intact E-boxes in the promoters of representative detoxification genes are important for the regulation of their expression, with SlituCLK, SlituBMAL1 acting as transcriptional promoters and SlituPER and SlituCWO as inhibitors (Zhang et al., 2021). Therefore, it might be speculated that in *S. litura* larvae the circadian system exerts a protective effect by maintaining a clear temporal separation between night feeding and daytime digestion/detoxification processes (Zhang et al., 2021).

It is important to note that the *S. litura* studies open the way for possible applications in agricultural pest control, as Zhang et al. (2021) demonstrated in *S. litura* larvae that topical treatments with insecticides were more efficient in inducing mortality when applied during nighttime than daytime. These findings are particularly relevant considering that crop management is evolving rapidly towards “precision agriculture” that supports management decisions for profitable and sustainable agricultural production. Automated systems and drones may also permit the distribution of pesticides when they are most effective against their target species. However, as species-specific differences are expected, preliminary studies of the larval circadian system in target pest Lepidoptera will enable specific and effective “chronobiological treatments,” limiting the spread of toxic insecticides in the environment.

## Conclusion

To date, the circadian clock has been examined in a relatively small number of species compared to the multitude of extant Lepidoptera. Nevertheless, the studies performed so far indicate the ecological relevance of the lepidopteran circadian system, as it influences the fitness of a single organism and in turn impacts the biodiversity of an ecosystem. It is easy to predict that the technological advances in Next Generation Omic technologies, as well as genome editing methodologies will facilitate the study and manipulation of model and non-model species. When applied to lepidopteran chronobiology, these methodologies will expand our current knowledge on the role of the circadian clocks in these organisms when considered in their natural habitat. Similar approaches will also be useful for the systematic study of species with economic importance such as *B. mori*, to understand whether manipulating the circadian clock can improve both rearing conditions and/or silk production efficiency. It is worthy to mention that Bm*per* KD lines exhibited a reduction in developmental time which did not affect silk productivity parameters (Sandrelli et al., 2007). Thus, the employment of genome editing techniques which generated Bm*per* and Bm*tim* KO *B. mori* mutants (Ikeda et al., 2019; Nartey et al., 2020) for producing a battery of silkworm lines carrying mutations in different clock genes might permit an efficient evaluation of these aspects in *B. mori*.

## Author Contributions

FS and DB: manuscript organization. FS, SC, DB, and AS: writing and editing. All authors contributed to the article and approved the submitted version.

## Funding

This project has received funding from the European Union’s Horizon 2020 research and innovation programme under the Marie Sklodowska-Curie grant agreement No. 765937 (CINCHRON).

## Conflict of Interest

The authors declare that the research was conducted in the absence of any commercial or financial relationships that could be construed as a potential conflict of interest.

## Publisher’s Note

All claims expressed in this article are solely those of the authors and do not necessarily represent those of their affiliated organizations, or those of the publisher, the editors and the reviewers. Any product that may be evaluated in this article, or claim that may be made by its manufacturer, is not guaranteed or endorsed by the publisher.
